# Unveiling the nexus between environmental exposures and testicular damages: revelations from autophagy and oxidative stress imbalance

**DOI:** 10.1038/s41420-025-02543-4

**Published:** 2025-05-29

**Authors:** Xiuwen Kong, Xinda Wang, Qiushi Xia, Qingqing Hu, Wenqian Yu, Qiuru Huang, Jiaxin Li, Chenyu Wang, Ziwen Lin, Yiheng Liu, Yujuan Qi, Xiaofang Tan, Bo Zheng, Jun Yu

**Affiliations:** 1 Institute of Reproductive Medicine, Jiangsu Province Key Laboratory in University for Inflammation and Molecular Drug Target, Medical School of Nantong University, Nantong University, Nantong, 226001 China; 2 Reproductive medicine Center, Affiliated Maternity and Child Health Care Hospital of Nantong University, Nantong, 226001 China; 3 Clinical Center of Reproductive Medicine, Xuzhou Central Hospital, Southeast University Affiliated Xuzhou Central Hospital, Xuzhou Clinical School of Xuzhou Medical University, Xuzhou, 221000 China; 4 State Key Laboratory of Reproductive Medicine and Offspring Health, Center for Reproduction and Genetics, The Affiliated Suzhou Hospital of Nanjing Medical University, Suzhou Municipal Hospital, Gusu School, Nanjing Medical University, Suzhou, 215002 China

**Keywords:** Spermatogenesis, Infertility

## Abstract

Recent evidence consolidates the deleterious impact of environmental exposure on testicular damage. Environmental exposures can instigate testicular toxicity, causing damage to the Sertoli-Sertoli cell-mediated blood-testis barrier (BTB) integrity, alterations in hormone levels orchestrated by aberrant Leydig cells, and disruption of spermatogenesis. Despite diverse study designs and methodologies, a consensus is emerging on how environmental factors induce oxidative stress by elevating ROS levels, affecting autophagy through pathways such as the ROS-mediated mTOR signaling pathway, ultimately culminating in testicular damage. This review synthesizes existing literature on how environmental exposures, including metals, air pollutants, industrial contaminants, and pesticides, disturb testicular homeostasis via autophagy-mediated oxidative stress, highlighting recent significant advancements. It also explores interventions like antioxidant support and autophagy regulation to alleviate testicular damage. These findings underscore the importance of elucidating the mechanisms of autophagy influenced by environmental exposures in disrupting the equilibrium of oxidative stress, identifying potential drug targets, and establishing a groundwork for enhancing future treatments and clinical management of testicular injuries.

## Facts

- An increase in male reproductive disorders characterized by diminished sperm quality and quantity, attributing these patterns to environmental exposure.
- Diverse environmental contaminants have the potential to interfere with spermatogenesis by adjusting cellular autophagy pathways, resulting in testicular harm.
- Mitigation tactics for testicular damage primarily involve antioxidative mechanisms and anti-autophagic methodologies.

## Introduction

The preservation of typical testicular function is meticulously overseen by genetic and environmental determinants [1–3], where persistent exposure to environmental stimuli can result in various testicular impairments [4]. Environmental exposure-triggered testicular harm primarily encompasses the compromise of Sertoli-Sertoli-cell-mediated blood-testis barrier (BTB) integrity, alterations in hormone levels orchestrated by aberrant Leydig cells, and disruption of spermatogenesis [5–8]. Environmental exposure impacts spermatogenesis, leading to the formation of vacuoles in seminiferous tubules, depletion of testicular germ cells, a significant decrease in sperm count, an increase in reactive oxygen species (ROS), culminating in apoptosis and irregularities in spermatogenesis [9–12].

**Fig. 1:**
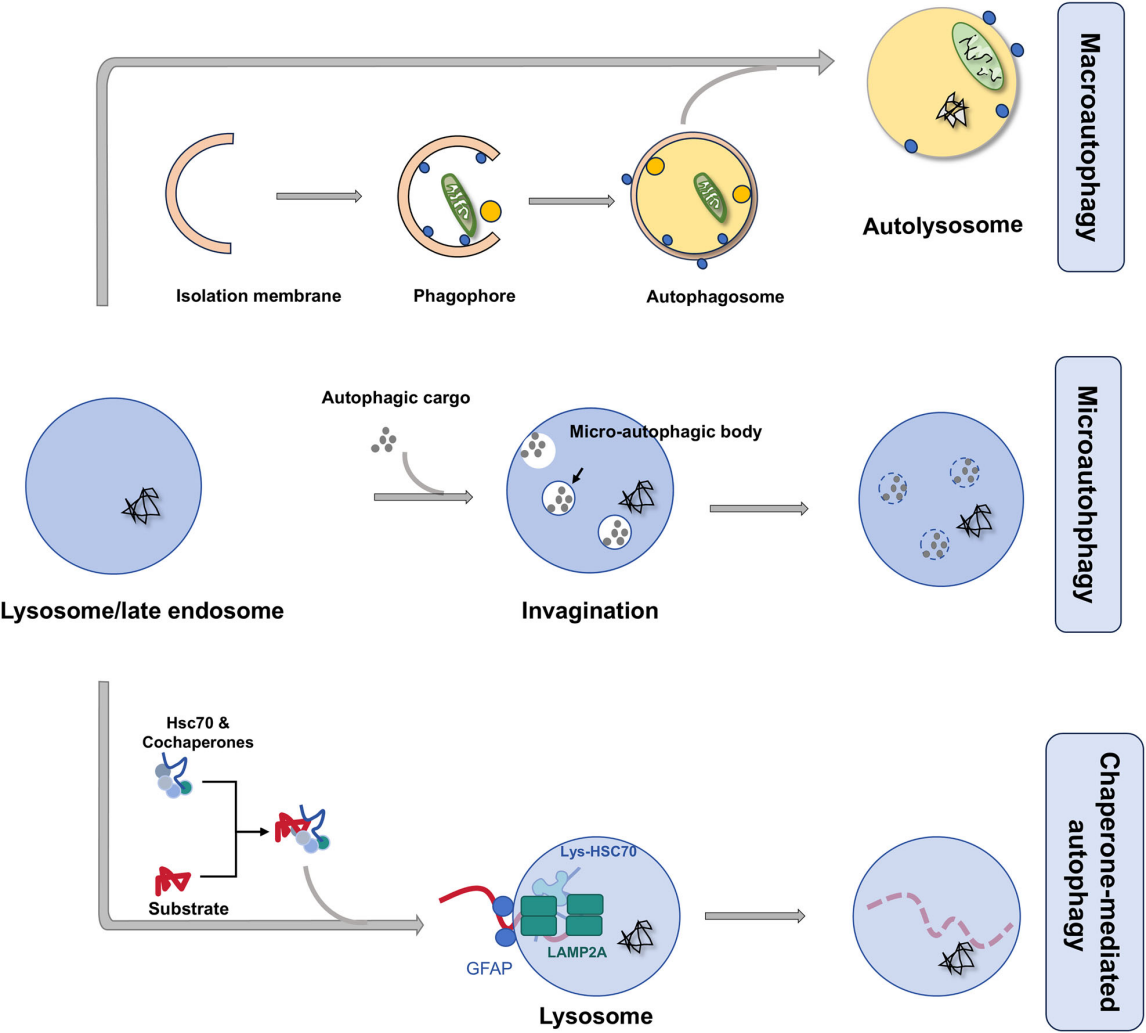
Depiction of the three primary autophagic mechanisms. Macroautophagy entails the generation of a phagophore that envelops the cargo, culminating in the formation of an autophagosome. This entity subsequently merges with a lysosome to create an autolysosome where degradation transpires. Microautophagy is portrayed by the direct engulfment of the substrate via the invagination of lysosomal or late endosomal membranes. Chaperone-mediated autophagy involves the recognition by the lysosome-associated receptor LAMP2A of specific cytosolic proteins harboring a KFERQ-like motif. The chaperone heat shock cognate protein 70 (Hsc70) aids in the translocation of these proteins into the lysosome for degradation.

Autophagy, an indispensable pathway for degradation and recycling, plays a vital role in maintaining cellular equilibrium, determining cell destiny, and fostering reproductive development [13]. It comprises three categories: macroautophagy, microautophagy, and chaperone-mediated autophagy, among which macroautophagy represents a major form [14] (Fig. 1). Accumulating evidence underscores the pivotal role of autophagy in diverse cellular processes within the male reproductive system. During spermatogenesis, autophagy is essential for both the formation of critical structures and the degradation of specific components, ensuring successful spermatid development [15]. In the autophagic process, the preautophagosome isolation membrane encapsulates protein aggregates or damaged organelles, leading to autophagosome formation, followed by fusion with the lysosome for cargo degradation by lysosomal enzymes [16]. The principal autophagic process revolves around autophagosome formation, regulated by an array of autophagy-related genes and pivotal signaling pathways, which include the mammalian target of rapamycin complex 1 (mTORC1), the serine/threonine-protein kinase ULK1/2, Beclin 1, autophagy proteins, class III phosphatidylinositol 3-kinases (PI3K), and microtubule-associated protein 1 A/1B-light chain 3-I/II (LC3-I/II) [17].

**Fig. 2:**
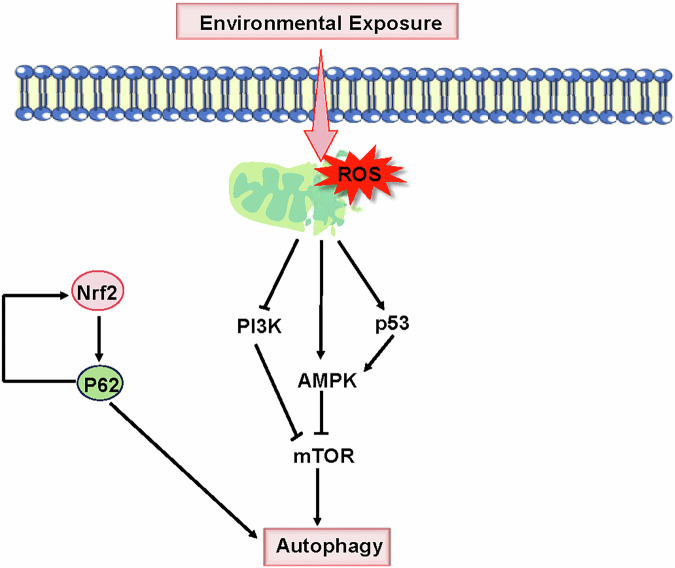
Principal pathways governing autophagy regulation in response to environmental stimuli. Exposure to environmental stressors induces the production of reactive oxygen species (ROS), thereby impacting autophagy through mTOR-dependent routes. Autophagy activation is achieved by inhibiting the PI3K-AKT-mTOR axis or by stimulating AMPK to suppress the mTOR signaling pathway. Moreover, p53 instigates AMPK activation and can lead to mTOR inhibition. The antioxidant factor Nrf2 and the autophagy-associated protein p62 mutually enhance each other’s expression.

Various endogenous sources like mitochondria, peroxisomes, and phagocytic cells, along with exogenous factors such as pollution, UV exposure, xenobiotic compounds, and cigarette smoke, can generate reactive oxygen and nitrogen species [18]. The generation of ROS in male germ cells, exceeding antioxidant defenses, leads to oxidative stress, triggering apoptosis, autophagy, and DNA damage, all of which are critical factors contributing to male infertility [19]. During spermatogenesis, the mitochondrial count diminishes to a range of 20-80 within the mature spermatozoon, and this limited mitochondrial population plays a crucial role in male fertility, as any disturbance in structures located at the tail of spermatids can impair fertility [20]. Mitochondria, being the primary sites of ROS production, possess the ability to regulate and modulate autophagy [21]. Mitophagy, responsible for mitochondrial quality control, involves the engulfment of dysfunctional mitochondria by autophagosomes, followed by lysosomal digestion post-fusion [22]. Diverse mechanisms are utilized by cells for mitophagy, particularly through the PINK1/Parkin partnership for depolarized mitochondria turnover, stress-induced BCL2 interacting protein 3 (BNIP3), BCL2 interacting protein 3 like (BNIP3L), and FUN14 domain containing 1 (FUNDC1) molecular adaptors that directly interact with the LC3 protein to facilitate mitophagy [23]. It is hypothesized that impaired mitochondria release increased ROS level, which in turn influence autophagy through mTOR-dependent pathways in the cytoplasm, either activating autophagy by inhibiting the PI3K-AKT-mTOR axis or by stimulating AMPK to suppress the mTOR signaling pathway [16, 24–26]. Furthermore, p53 triggers AMPK activation, leading to subsequent mTOR inhibition [16] (Fig. 2).

Oxidative stress denotes an increase in intracellular ROS levels, resulting in harm to lipids, proteins, and DNA [27, 28]. In response to oxidative stress, NFE2 like bZIP transcription factor 2 (Nrf2) regulates antioxidant defenses by recognizing and binding to antioxidant response elements (ARE) sequences [29]. Keap1 inhibits nuclear activation of ARE by Nrf2, sequestering Nrf2 in the cytoplasm through the Keap1-Cul3 complex for ubiquitin-proteasome degradation, with oxidants or electrophiles modifying Keap1 to prevent Nrf2 ubiquitination and promote its nuclear translocation [29, 30]. Nrf2 binds to the ARE motif in the p62 promoter, enhancing p62 mRNA expression to stimulate cytoplasmic autophagy as a post-transcriptional regulatory mechanism, while p62 reciprocally boosts NRF2 transcription [31] (Fig. 2). Studies have demonstrated that an excess of p62 or autophagy deficiency disrupts the Nrf2 and Keap1 interaction, resulting in Nrf2 stabilization and subsequent transcriptional activation of Nrf2 target genes [32]. Additionally, the FUNDC1 phosphatase PGAM family member 5, mitochondrial serine/threonine protein phosphatase (PGAM5) serves as a Keap substrate, suggesting a connection between PGAM5 ubiquitination and degradation, as well as FUNDC1 dephosphorylation, culminating in the initiation of FUNDC1-dependent mitophagy [29].

Recent epidemiological data demonstrate a rise in male reproductive disorders with decreased sperm quality and quantity, linking these trends to environmental exposure based on increasing evidence from human and animal studies [33, 34]. Human activities like mining, industrial discharges, and electronic waste recycling have escalated metal contamination, with harmful metals gathering in vital organs such as the testis, heart, liver, kidneys, and brain [35, 36]. Predominantly comprising carbonaceous particulate matter (PM) and gases like NO2, SO2, CO, and volatile organic compounds (VOCs), air pollution stems from varied sources like residential energy consumption, industrial emissions, and vehicular exhaust [37]. Synthetic chemicals pose risks to post-polymerization, with leachable monomers or additives like bisphenols potentially diffusing into the environment, leading to inadvertent exposure [38]. Pesticides, omnipresent in our environment, exert deleterious effects on human health, potentially contributing to male infertility by adversely affecting non-target organs and reproductive health upon prolonged exposure [39, 40].

Research suggests that various environmental pollutants can disrupt spermatogenesis by modulating cellular autophagy pathways, leading to testicular damage. To better understand the subject, this review used many keywords, including ‘testicular damage’, ‘ROS’, ‘oxidative stress’, ‘apoptosis’, ‘autophagy’, and ‘male infertility’, along with related keywords in major online databases. Therefore, this paper systematically reviews the research progress on how environmental exposures, such as metals, air pollutants, industrial contaminants, and pesticides disrupt testicular homeostasis via autophagy-mediated oxidative stress (Table 1).

**Table 1:** Synopsis of key discoveries: Testicular damage due to environmental exposures involves autophagy and oxidative stress disruption.

Classification	Environmental exposure		Animal strains	Exposure concentrations	Exposure routes	Male reproductive toxicity	Potential targets
Metal	Cadmium		C57BL/6 mice	50 mg/L Cd for 3 months	Orally administration	Sperm quality decrease Nuclear depression and deformation Mitochondria damage Autophagy increase	Autophagy: LAMP2, LC3, ATG7, ATG5, Beclin1, P62
	Cadmium		SD rats	0.2 mg/kg, 0.4 mg/kg, 0.8 mg/kg CdCl_2_ for 5 weeks	Intraperitoneal injection	Sperm quality decrease Testicular architecture damage Testicular cell apoptosis increase Oxidative stress imbalance Autophagy increase	Autophagy: LC3, P62, Beclin-1
	Hexavalent chromium		Wistar rats	2, 4, and 6 mg/kg body weight K2Cr2O7 for 5 weeks	Intraperitoneal injection	Sperm quality decrease Testicular architecture damage Mitochondria damage DNA damage Oxidative stress imbalance Apoptosis increase Autophagy increase	Autophagy: LC3, P62, Beclin-1 Oxidative stress: Nrf2, Mfn2, HO-1, NQO1, Sirt1, PGC-1α
	Metallic nanomaterials	Gold nanoparticles	BALB/c mice	0.17 mg/kg and 0.5 mg/kg AuNPs for 14 days	Intravenous injection	Plasma testosterone levels reduce Sperm quality decrease Oxidative stress imbalance Autophagy increase	Oxidative stress: IL-1, IL-6, IL-8, TNF-a Autophagy: autophagosome related factors
		Copper nanoparticles	SD rats	44, 88, and 175 mg/kg/day nanocopper for 28 days	Oral gavage	Sperm quality decrease Sex hormones decrease Testicular architecture damage Autophagy increase Oxidative stress imbalance	Autophagy: LC3, ATG5, ATG7, ATG12, Beclin1, P62, AKT/mTOR signaling pathway
Air pollutant	Particulate matter	PM2.5	SD rats	9 mg/kg.b.w, 24 mg/kg.b.w PM2.5 for 7 weeks	Intratracheally instilled	Sperm quality decrease Testicular architecture damage	Oxidative stress: PI3K/Akt signaling pathway
	Tobacco	Nicotine	SD rats	0.6 mg/kg nicotine for 28 days	Intraperitoneal injection	Testicular artichitecture damage Spermatogenesis suppress Sperm quality decrease Oxidative stress imbalance	Oxidative stress: SIRT6/Bmal1 regulatory pathway
	Aldehydes	Formaldehyde	SD rats	0.5, 5 and 10 mg/m³ formaldehyde 8 h/d for 4 weeks	Inhalation	Testicular seminiferous tubules atrophy Spermatogenic cells decreased Autophagosome increase Damaged and abnormally shaped ER and mitochondria	Oxidative stress: mTOR pathway Autophagy: LC3
Synthetic chemicals	Nano-plastics		BALB/c mice	100 nm, 5 mg/kg/day or 50 mg/kg/day polystyrene NPs for 30 days	Oral gavage	Sperm quality decrease Testicular architecture damage Apoptosis increase damages the integrity of BTB Impaired autophagy	Autophagy: LC3 and P62
	Bisphenol A		BALB/c mice	5, 20, 50 mg/kg BPA for 30 days	Intraperitoneal injection	Sperm quality decrease Oxidative stress imbalance Endocrine disorder Apoptosis increase Autophagic flux blocked Autophagosome accumulation	Autophagy: LC3, ATG7, P62 Oxidative stress: GPX5, SOD1, SOD2, and CAT
	Di-(2-ethylhexyl) phthalate		SD rats	250 and 500 mg/kg DEHP from postnatal day (PND) 1 to PND 35	Intragastric administration	Testicular architecture damage Oxidative stress imbalance Autophagosome accumulation	Autophagy: autophagosome LC3 and P62 Oxidative stress: HO-1 and SOD
	Perfluorooctane sulfonate		ICR mice	0.5, 5, and 10 mg/kg/day PFOS for 5 weeks	Gavage	Sperm quality decrease Serum testosterone levels decrease Testicular architecture damage Autophagy increase	Autophagy: PI3K/AKT/mTOR pathway, P62, ATG5, ATG7, and LC3
	Perfluorooctanoic acid		BABL/c mice	1.25, 5, and 20 mg/kg/d PFOA for 28 days	Gavage	Testicular architecture damage Autophagic flux blocked Autophagosome accumulation	Autophag: LC3, Beclin1 and P62
	Perfluorooctanoic acid		Kunming mice	2.5, 5 or 10 mg/kg/day PFOA for 14 days	Orally administrated	Sperm quality decrease Testicular architecture damage Oxidative stress imbalance Apoptosis increase	Oxidative stress: NRF2, P53
	4-Nonylphenol		SD rats	25, 50 or 100 mg/kg 4-NP body weight for 20 days	Intraperitoneal injection	Impaired spermatogenesis and sperm function Sex hormones deficiency Oxidative stress imbalance Apoptosis increase Autophagy increase	Oxidative stress: P53 Autophagy: Beclin-1,LC3, ATG3, ATG5, ATG7, ATG12, AMPK-mTOR-p70S6K/4EBP1 signaling pathway
	Acrylamide		SD rats	40 mg/kg Acrylamide for 10 days	Intraperitoneal injection	Testicular architecture damage Sex hormones decrease Oxidative stress imbalance Apoptosis increase	Oxidative stress: MAPK14 and P53
Pesticide	Insecticides	Cyfluthrin	Wistar rats	6.25, 12.5, 25 mg/kg bw Cyfluthrin for 4 weeks	Gavage	Testicular architecture damage Autophagy increase Oxidative stress imbalance	Autophagy: AMPK/mTOR/P70S6K signal pathway
	Insecticides	Avermectin/ Abamectin	SD rats	1 mg/kg/day ABM for 28 days	Orally administration	Oxidative stress imbalance ER stress Autophagy increase Apoptosis increase	Autophagy: MAPK14/IL-6/JAK2/STAT3 pathway
	Herbicides	Fluorochloridone	C57BL/6 mice	3, 15, 75, 375 mg/kg/day Fluorochloridone for 28 days	Gavage	Sperm quality decrease Autophagy increase	Autophagy: LC3, Beclin-1 and P62, AKT/mTOR signaling pathways
	Fungicides	Thiram	Kunming mice	30, 60, 120 mg/kg Thiram for 21 days	Oral gavage	Testicular architecture damage Autophagy increase Oxidative stress imbalance	Autophagy: mTOR and P62

## Metal

### Cadmium

A primary source of exposure for the general population is cadmium, which is widespread in numerous human food items due to its high soil-to-plant transfer rates [41]. The current global standards for tolerable intake and acceptable excretion of cadmium are set at 0.83 µg/kg body weight per day and 5.24 µg/g creatinine, respectively [42]. Various studies have highlighted the heightened susceptibility of mammalian testes to cadmium, leading to toxicity in male reproductive organs, specifically affecting the testicles and sperm parameters [43]. Within the Sertoli cells of the testis, cadmium has been observed to notably elevate the levels of autophagy markers such as LC3, p62, ATG7, Beclin-1, and ATG5, along with the lysosomal membrane protein LAMP2 [44]. Excessive autophagy activation can culminate in cell death under conditions of oxidative stress and metal toxicity [44]. The mTOR pathway, functioning as a central checkpoint that negatively modulates autophagy, assumes a critical role upstream of autophagy during oxidative stress, implying that the mTOR signaling pathway may represent a primary conduit through which cadmium amplifies autophagy and induces reproductive toxicity [45]. This underscores the significance of the ROS signal in governing the cadmium-disrupted autophagy process in Leydig cells [46]. Notably, emerging reports have suggested that exposure to cadmium in testicular tissue can instigate defective autophagy flux, despite the upregulation of several autophagy-related factors, including ATG3, ATG5, p62/sequestosome-1 (SQSTM1), and Beclin 1 [47].

### Arsenic

Arsenic, a naturally occurring element in the earth’s crust, is found as a contaminant in a diverse range of metal ores [48]. This element poses a significant health hazard, with estimates indicating that over 100 million individuals worldwide are exposed to arsenic levels deemed carcinogenic, primarily through the consumption of drinking water taken from arsenic-contaminated aquifers [49, 50]. Arsenic compounds As (III) and As (V) are classified as non-threshold Class I carcinogens with acute toxicities ranging from 15 to 42 mg/kg body mass. In contrast, simple methylated arsenicals exhibit intermediate toxicity levels, while the tetraalkylated compound arsenobetaine (AB), a common dietary source of arsenic, is considered non-toxic with a lethal dose 50% (LD50) surpassing 10,000 mg/kg body weight, primarily excreted intact in urine by humans [51]. Studies have shown that arsenic, known to trigger ROS production, disrupts spermatogenesis by impeding spermatid elongation, notably impacting semen quality and prompting endocrine dysfunction [52, 53]. Moreover, arsenic exposure has been linked to a remarkable increase in protein expression levels of Beclin-1, LC3, ATG7 and p62, and knockdown of *beclin-1* has been shown to attenuate the alterations induced by arsenic treatment in MLTC-1 cells [54]. Furthermore, arsenic accumulation in testes, leading to increased oxidative stress markers like malondialdehyde (MDA), superoxide dismutase (SOD), and methionine sulfoxide reductases (MsrA), may effectively trigger autophagy and apoptosis processes [55].

### Copper

Essential for maintaining overall health and fertility, copper can however exhibit toxicity when present in excessive amounts, with detrimental effects on male fertility [56]. Research indicates correlations between copper levels in seminal plasma and sperm quality parameters like motility, viability, and morphology [57]. Studies in chicken testes show increased expression of genes related to mitochondrial fission alongside decreased levels of fusion-related genes [58]. Copper exposure in mouse testes induces oxidative stress, characterized by increased ROS, MDA, and lactate dehydrogenase (LDH), coupled with reduced catalase (CAT) activity and glutathione (GSH) levels [59]. This oxidative environment leads to mitochondrial dysfunction, as indicated by lowered transmembrane potential and ATP levels, with upregulated autophagy-related genes and proteins pointing towards copper-triggered cell death and autophagy mediated by oxidative stress-induced mitochondrial dysfunction [59]. Moreover, copper overload in *Drosophila* also impacts testicular aging, highlighting the interplay between copper overload, long non-coding RNAs (lncRNAs), and the induction of cuproptosis and ferroptosis pathways through the mitochondrial tricarboxylic acid (TCA) cycle [56].

### Hexavalent chromium

Hexavalent chromium emerges as the most potent carcinogen among hazardous heavy metal(loid)s contaminants in agricultural soil, water, and air, commonly associated with activities like metallurgic industries, tanneries, paint manufacturing, and petroleum refineries [60]. As per the North Carolina Health Department in the United States, the concentration of 0.07 µg/L equates to a 1-in-1-million lifetime cancer risk [61]. Consequently, the numerous instances of surpassing this threshold suggest a potentially significant portion of the population at risk. Treatment with hexavalent chromium disrupts spermatogenesis, resulting in the accumulation of prematurely released spermatocytes, spermatids, and uni- and multinucleate giant cells within the seminiferous tubules [62]. Studies conducted on rat testes have revealed that hexavalent chromium suppresses the Sirtuin1 (SIRT1)/peroxisome proliferator-activated receptor gamma coactivator-1alpha (PGC-1alpha) pathway, leading to mitochondrial dynamics disorder characterized by elevated mitochondrial division and inhibited mitochondrial fusion [63]. Furthermore, downregulation of the downstream effector Nrf2 from Sirt1 exacerbates oxidative stress, leading to mitochondrial dynamics disorder and Nrf2 inhibition, ultimately contributing to abnormal testicular mitochondrial dynamics and enhancing apoptosis and autophagy [63].

### Metallic nanomaterials (NMs)

Metallic NMs are employed in industrial contexts for various applications that often result in environmental release. These applications encompass antimicrobial coatings, fuel cells, water electrolysis, air and water purification, as well as biomedical imaging contrast agents [64]. Numerous nanoparticles (NP) have been identified to adversely impact spermatogenesis [65]. Gold nanoparticles (AuNPs), specifically noted for their catalytic attributes and biomedical uses, have sparked a growing interest in comprehending the potential toxic repercussions of such particles [66–68]. Liu et al., demonstrated that AuNPs (5 nm) can penetrate into the endosomes/lysosomes in Leydig cells, induce autophagosome formation, elevate ROS production, and interfere with the cell cycle in the S phase, thereby inducing concentration-dependent cytotoxicity and DNA impairment [69]. Meanwhile, biogenic nanocopper (BNC) agents exhibit robust anticancer, antimicrobial, and antiparasitic properties, and due to their minimal impact on normal cells, they are favored for treating various ailments [70]. Exposure to nanocopper increases autophagy-related factors like LC3, ATG5, ATG7, ATG12, and Beclin-1, while reducing p62 levels, linking nanocopper-induced damage in testicular tissues and spermatogenesis to cell apoptosis and autophagy via Akt/mTOR signaling and oxidative stress [71]. Silver nanoparticles (AgNPs) find utility in a broad array of products, spanning electronics, biosensors, textiles, the food industry, coatings, sunscreens, cosmetics, and medical devices [72]. AgNPs as stressors elevate ROS production, potentially upregulating p53, Bax, and Caspase-3 in response to stress factors in testicular tissues, leading to programmed cell death [73].

## Air pollutant

### PM

As a prominent constituent of air pollution, the health implications of PM2.5 (aerodynamic diameter ≤2.5 μm) represent a critical public health concern [74]. Research reveals a notable 2.0% decline in fertility rates per 10 mg/m³ rise in PM2.5 levels, as illustrated by PM2.5 mapping data in China [75]. Subsequent evaluation of testicular impact post-PM2.5 exposure demonstrated a significant increase in abnormal sperm morphology rates in both low and high PM2.5 dosage groups compared to controls [76]. Oxidative stress emerges as a pivotal initiator of reproductive impairment induced by PM2.5, with its ability to escalate cellular oxidative stress through excessive ROS generation, culminating in cell demise [76, 77]. Following PM2.5 exposure, heightened expression levels of PI3K and pAKT were observed in Sertoli cells, suggesting a potential role of ROS as signaling molecules activating Nrf2-mediated defenses against PM2.5-induced oxidative stress via the PI3K/AKT pathway [78, 79]. Conversely, in GC-spg cells, PM2.5 exposure led to a significant reduction in PI3K phosphorylation at 5 μg/cm², with both PI3K and AKT phosphorylation diminishing at 20 μg/cm², accompanied by elevated autophagy activity [80]. Despite stable Beclin-1 and p-p62 levels, elevated ATG7 and LC3 expression suggested PM2.5 could promote autophagosome formation [80].

### Tobacco

Nicotine, a toxic alkaloid from tobacco plants, has been detected in various water sources, including surface waters, ground waters, industrial waste waters, and bottled waters [81, 82]. Studies show that ingestion of nicotine gums up to 6 mg/kg can induce intoxication symptoms in humans without causing fatality [83]. In rats, nicotine exposure has dose-dependently reduced sperm count and motility, while inducing seminiferous tubule and spermatogenic disruptions in the testes, possibly via excessive mitochondrial fusion triggering oxidative stress [84, 85]. Chronic low-dose nicotine exposure is implicated in oxidative stress through free radical-mediated lipid peroxidation (LPO) and protein oxidation in the testes and prostate, potentially due to altered mitochondrial dynamics favoring fusion over mitophagy [85, 86]. Moreover, nicotine treatment marginally increased autophagosome formation but hindered their fusion with lysosomes, accompanied by elevated LC3II/LC3I ratio and p62 levels, indicating impaired autophagic turnover in Leydig cells [87].

### Aldehydes

Formaldehyde (FA) is a prevalent environmental pollutant encountered in various settings such as outdoors, indoors, workplaces, and residences, primarily through inhalation exposure [88]. Evidence from human and animal studies suggests that FA exposure can induce reproductive toxicity, inhibiting spermatogenesis with seminiferous tubule degeneration, spermatogenic cell apoptosis, lowered testosterone levels, and disrupted testicular antioxidant defenses [89, 90]. FA exposure elevates ROS levels and reduces SOD and GSH activities, while increasing MDA in rat testes, indicating oxidative stress-induced testicular damage [91]. Moreover, FA exposure dose-dependently induces autophagy, evidenced by LC3-I/LC3-II conversion and elevated LC3-II expression in testes [90]. Recent research suggests that FA exposure suppresses mTOR expression in testicular tissue, correlating with increased testicular autophagy levels in an mTOR-dependent manner [89].

Acrolein exposure is prevalent, originating from sources such as cigarette smoke, industrial pollution, and other environmental exposures that are known to increase the production of ROS [92]. Acrolein, one of the most reactive lipid aldehydes generated during the LPO process in oxidatively stressed spermatozoa, inhibits sperm motility and escalates ROS production, LPO, oxidative DNA damage, and Caspase activation [93]. Exposure of mouse Sertoli cells to acrolein resulted in a concentration-dependent elevation in cell mortality, with the deleterious impact associated with oxidative stress via p38 activation [94]. In Leydig cells, acrolein induces toxic ROS production and reduces superoxide dismutase activity, leading to increased lipid oxidation reflected by elevated MDA level, thereby initiating oxidative stress [95]. This exposure also initiates AKT protein expression under oxidative stress, triggering early-stage autophagy via the PI3K/AKT/mTOR pathway, which progresses to activate apoptosis-related pathways, culminating in programmed cell death in Leydig cells [95].

## Synthetic chemicals

### Nano-plastics

Nano-plastics, originating as byproducts from the degradation and manufacturing processes of plastic items, possess colloidal properties and range in size from 1 to 1000 nanometers (nm) [96]. Individuals inadvertently consume or inhale nano-plastics face potential health risks, with an estimated annual exposure to 39,000–52,000 nano-plastics [97]. Studies have demonstrated that when mice are orally administered polystyrene nanoparticles (PS-NPs) measuring an average size of 38.92 nm, at doses of 1, 3, 6, and 10 mg/kg/day for 35 days, these NP accumulate in various organs including the testes, intestines, liver, kidney, and brain, with the highest accumulation observed in the testes [98]. Concurrently, another study observed a notable reduction in sperm concentration was observed in groups exposed to PS-NPs at equivalent concentrations and durations, with a notably higher percentage of sperm showing abnormal morphology compared to the control group [99]. Numerous studies have indicated that ROS overproduction serves as the primary event triggering male reproductive toxicity induced by nanoplastics in mammals, subsequently instigating oxidative stress [100]. The surge in ROS initiates multiple cascading events at various levels, encompassing cellular oxidative stress, mitochondrial dysfunction, sperm DNA damage, endoplasmic reticulum (ER) stress, apoptosis, and autophagy of testicular cells [100]. ROS can compromise the integrity of mitochondrial DNA (mtDNA), establishing a detrimental cycle where compromised mitochondria, due to oxidized mtDNA, become dysfunctional, leading to excessive ROS production, further exacerbating mitochondrial impairment and ultimately resulting in severe nuclear DNA damage and cell demise [21]. Notably, nano-plastics significantly augment the expression of the autophagy biomarkers LC3-II and p62 while concurrently suppressing mTORC expression, indicating that nano-plastics could induce excessive autophagy by modulating mTORC signaling in spermatocyte cells [101]. Following exposure to PS-NPs in spermatocyte cells, a significant decrease in Nrf2 and HO-1 expression was noted, leading to the activation of mitochondrial apoptosis and autophagy pathways [100]. When autophagy is initiated by ROS, p62 undergoes degradation, disrupting the feedforward loop linking Nrf2 and p62, leading to a direct decrease in antioxidant capacity and an increase in ROS levels [102].

### Bisphenol A (BPA)

BPA, a commonly used plasticizer, is readily absorbed by both animals and humans, exerting toxic effects on a range of tissues such as the liver, intestine, heart, kidney, testes, and ovary [103, 104]. The estimated daily intake of BPA for adults via drinking tap water is 148 ng/day [105]. BPA induces a range of testicular impairments, affecting seminiferous tubules, sperm quality, germ cells, and the BTB, intricately linked to molecular processes involving ROS, apoptosis, and autophagy [106]. Exposure to BPA induces oxidative stress in the testicular niche cells, as evidenced by elevated levels of MDA and reduced SOD activity, consequently heightening ROS levels in vitro [106]. Exposure to BPA activates the AKT pathway and inhibits the mTOR pathway, leading to concurrent apoptosis and autophagy in adolescent testes [107]. High-dose exposure results in increased autophagosomes in seminiferous epithelial cells, displaying irregular shapes with cytoplasm, damaged ER, and abnormal mitochondria, surrounded by secondary lysosomes, suggesting active phagocytosis post-BPA exposure [107]. Moreover, p62, a Keap1-Nrf2 pathway component, is degraded through autophagy, disrupting the feedback loop, resulting in reduced antioxidant capacity and increased ROS levels [102].

### Di-(2-ethylhexyl) phthalate (DEHP)

Phthalate esters (PAEs) are commonly utilized organic chemicals as plasticizers in various industrial applications [108]. Among these, DEHP is extensively employed in plastics, rubber, adhesives, and other materials, yet concerns arise due to its leakage into greenhouse vegetables, dust, and medical equipment, leading to significant acute exposure levels [109]. Notably, DEHP exposure results in decreased serum testosterone levels, potentially harming Leydig cell function [110]. Upon entry into the body, DEHP induces ROS overproduction, triggering oxidative stress marked by elevated MDA and reduced GSH levels, implicated in apoptosis and autophagy of Leydig cells [110]. DEHP exposure also disrupts Sertoli cell function and compromises the BTB integrity [111]. Furthermore, DEHP exposure elevates ROS levels and simultaneously increases the number of autophagosomes by impairing autophagy degradation [111]. On the other hand, DEHP can also induce testicular injury through the excessive generation of ROS in immature testes and DEHP-triggered autophagy may lead to the hyperactivation of the NLRP3 inflammasome, resulting in germ cell impairment via the ROS/mTOR/NLRP3 pathway [112].

### Perfluorooctane sulfonate (PFOS) and perfluorooctanoic acid (PFOA)

PFOS and PFOA have found application in numerous fields, including waxes and polishes, fabric protection, stain repellents for textiles and leather and food packaging coatings that resist oil [113]. PFOS and PFOA exposures can occur through multiple pathways, including consumption from food packaging, food migration, or direct ingestion [114]. Studies on male reproductive toxicity have indicated that PFOA and PFOS can lead to reduced serum testosterone levels, testes weights, increased abnormal sperm counts, and disruption of the BTB in rodent models [115–118]. PFOA and PFOS exposures have been linked to the ROS generation via oxidative stress imbalance in mammalian cells [119]. PFOS exerts its reproductive toxicity by disrupting the BTB and also increases ROS production, leading to the decreased activity of the PI3K/AKT/mTOR pathway to induce autophagy in Sertoli cells [116]. Exposure to PFOA leads to elevated ROS production, heightened p53 levels inhibiting NRF2, resulting in testicular oxidative stress characterized by raised LPO, germ cell apoptosis, and diminished antioxidants in mouse testes [119]. Moreover, PFOA hinders autophagic breakdown by blocking autophagosome-lysosome fusion, linked to reduced α-SNAP expression, which reduces the levels of TJ-related proteins (Occludin and Claudin-11), GJ-related protein (connexin-43 or CX43), and ES-related proteins (N-cadherin and β-catenin) in Sertoli cells [120]. Within Leydig cells, PFOA diminishes LC3-II levels and elevates p62 levels in a dose-dependent manner, indicating a suppression of autophagosome formation, yet autophagy activation mitigates PFOA-triggered apoptosis, underscoring PFOA’s cell injury induction through autophagosome formation inhibition [115].

### 4-Nonylphenol (4-NP)

4-NP, a persistent environmental contaminant within the alkylphenol group, is widely employed in various products like detergents, lubricants, cosmetics, pesticides, plastics, paints, and wetting agents [121]. It is prevalent in food sources such as fish, animal tissues, milk, cereals, vegetables, and fruits due to its lipophilic nature and extended half-life, leading to bioaccumulation in aquatic organisms and humans [122]. Research on rodents indicates that 4-NP detrimentally affects male reproductive function, causing testicular apoptosis, seminiferous tubule degeneration, reduced testicular germ cell and Sertoli cell counts, sperm abnormalities, and diminished sperm quality, count, and viability [123]. Sertoli cells have been identified as targets of 4-NP, with studies showing its potential to induce apoptosis and autophagy in these cells upon early exposure [123]. 4-NP exposure triggers oxidative stress by increasing ROS and MDA levels while reducing SOD and CAT activities, culminating in ROS-induced AMPK activation that suppresses mTOR activity, boosts Beclin-1 expression, and enhances the LC3-II/LC3-I ratio, thereby initiating Sertoli cell autophagy potentially through the ROS-mediated AMPK-mTOR pathway [124, 125].

### Tributyltin chloride (TBTCL)

Extensively employed as a biocide in antifouling paints and agricultural products, TBTCL has caused environmental and marine pollution, with human exposure which mainly stems from tainted seafood consumption, fungicide use on crops, and potential contact with organotin-stabilized polyvinyl chloride in various products like food packaging, plastics, and water pipes [126]. Exposure to TBTCL elicits a cellular response marked by increased calcium levels that stimulate the ROS generation from mitochondrial ATP production systems, disrupting the ROS-GSH balance, causing oxidative stress, and ultimately leading to cell death in Sertoli-germ cell cocultures [127]. TBTCL exposure is also linked to decreased testosterone production, triggering ER stress and inhibiting autophagy flux, leading to apoptosis and cell cycle arrest in Leydig cells [128].

### Acrylamide

Acrylamide, a highly toxic compound utilized in plastics, paper production, dyes, and water treatment, is also a significant by-product formed during the high-temperature cooking of starchy foods, representing a primary source of human exposure estimated at around 1 μg/kg bw/day [129]. This environmental chemical exerts detrimental effects on biological systems, contributing to human infertility [130]. Farag et al., demonstrates that exposure to acrylamide can result in decreased sperm quality, testicular degeneration, epididymis weight loss, and disrupted steroidogenic signaling [131]. Studies further indicate that acrylamide exposure in rat testes leads to a notable increase in MDA level alongside reduced GSH level [132]. Subsequent to oxidative stress, acrylamide administration prompts a significant elevation in testicular AMPK gene expression and phosphorylated protein levels, which downregulate PI3K and mTOR, as well as pAKT content, ultimately instigating an autophagic apoptosis process in testes [133].

## Pesticide

Pesticides encompass a range of classifications, such as insecticides, herbicides, fungicides, rodenticides, acaricides, and fumigants [134]. One method of categorization is based on the LD50 value, which indicates the level of toxicity of a pesticide. Pesticides are stratified as highly toxic, moderately toxic, slightly toxic, or relatively nontoxic based on their LD50 values [135]. Exposure to pesticides can transpire through four primary pathways: oral, dermal, respiratory, and ocular exposure [136]. Prior studies have revealed notable correlations between human exposure to pesticide and decreased sperm quality [137]. This review delves into the realm of insecticides, herbicides, and fungicides.

### Insecticides

Globally recognized as one of the most potent pyrethroid insecticides, lambda cyhalothrin, also known as cyhalothrin, exhibits a wide range of applications and is characterized by its potency and fast action as both an insecticide and acaricide [138]. Concentrations of lambda-cyhalothrin in surface water have been documented to range from 0.35 to 0.80 μg/L, heightening the potential risk of human exposure to this substance [138]. Lambda-cyhalothrin exposure may result in the abnormal ROS accumulation, potentially leading to oxidative stress [139], which can directly damage DNA by oxidizing nucleoside groups, including the formation of 8-oxoguanine [138]. Elevated oxidative stress levels can trigger mitochondrial dysfunction during cyfluthrin-induced testicular injury, accompanied by decreased p62 levels at both protein and mRNA levels and a gradual increase in LC3 expression, indicating a potential elevation in autophagy levels [140].

The pyrethroid insecticide cypermethrin, which is extensively employed, holds the potential to provoke adverse endocrine-disrupting impacts on the male reproductive system [141]. Cypermethrin has been found to disrupt mitochondrial membrane integrity and slightly increase the levels of Sqstm1/p62 protein in the mitochondria of mouse Leydig and Sertoli cells, indicating its potential to impair mitochondrial function and inhibit mitophagy [142]. Owing to their lipophilic characteristics, pyrethroids like cypermethrin can accumulate in cellular membranes, fostering the ROS generation, which can induce oxidative harm in animals [143].

Avermectins represent broad-spectrum antiparasitic agents wildly used in agriculture and for the treatment of domestic animals [144]. Furthermore, multiple studies have illustrated the adverse impacts of avermectins on male fertility [145, 146]. Abamectin exposure at levels pertinent to both occupational and environmental settings has been associated with decreased sperm quality parameters, particularly a decline in sperm concentration [147]. Abamectin has been evidenced to instigate oxidative stress, eliciting ER stress, inflammation, apoptosis, and autophagy [148]. Oxidative stress is regarded as a pivotal element in the cytotoxicity induced by avermectins [149]. It has been postulated that exposure to avermectins prompts apoptosis and autophagy in Leydig cells by accumulating ROS, which orchestrates the suppression of the PI3K/AKT/mTOR signaling pathway [150].

Imidacloprid, known chemically as 1-(6-chloro-3-pyridylmethyl)-N-nitroimidazolidin-2-ylideneamine, is a systemic insecticide classified under the neonicotinoid group, designated by the World Health Organization (WHO) as a Class II hazardous pesticide due to its enduring presence and toxicity, it poses risks to ecosystems by potentially disrupting food chains and biogeochemical cycles [151]. Imidacloprid is known to elicit male reproductive toxicity in mammals [152]. The male reproductive organs are particularly vulnerable to the harmful impacts of ROS, with imidacloprid’s detrimental effects on the male rats’ reproductive system thought to stem from inducing oxidative stress in the testes [153]. Lysosomes and distinct autophagic vacuoles containing damaged mitochondria and other cellular organelles can be observed in Leydig cells exposed to 400 and 500 µM imidacloprid [152]. Additionally, a study elucidates that the oxidative stress induced by imidacloprid leads to mitochondrial dysfunction, subsequently activating the nuclear factor-kappa B (NF-κB)/c-Jun N-terminal kinase (JNK) pathway to modulate mitochondrial apoptosis and BNIP3-mediated mitophagy [154].

### Herbicides

Glyphosate functions as a broad-spectrum herbicide and stands as one of the extensively researched pesticides [155]. The Food and Agriculture Organization (FAO) has emphasized glyphosate’s potential toxicological risks from residue accumulation in the food chain, but stated that the risk of dietary exposure is unlikely if the maximum daily intake stays below 1 mg/kg of body weight [155]. Glyphosate exposure has been linked to male reproductive impairment through the disruption of the BTB, deterioration of sperm quality and seminal parameters, and inhibition of testosterone production [156]. Previous research on sperm has indicated that impaired spermatogenesis following glyphosate exposure is associated with oxidative stress induced by excessive ROS [157]. Glyphosate-induced dysfunction in TM3 cells, featuring abnormal mitochondria, disrupted dynamics, increased mitochondrial ROS production, decreased steroidogenic enzyme levels, and suppressed testosterone synthesis, was linked to heightened autophagic flux and mitophagy, the latter dependent on Parkin activation [158].

During the cultivation of various crops like winter wheat, potatoes, sunflowers, sugar canes and cotton, a pyrrolidone herbicide known for its selective properties, namely flurochloridone, is utilized to regulate a diverse range of broadleaf weeds and grasses [159]. Studies suggest that fluorochloridone may function as a potential endocrine disruptor, exerting adverse effects on the reproductive functions and hormonal equilibrium in male rats [160]. Fluorochloridone exposure has been associated with ROS buildup, mitochondrial dysfunction, and the initiation of cell apoptosis in Sertoli cells [161]. In live organisms, fluorochloridone promotes the formation of autophagosomes and elevates the levels of LC3II/LC3I, Beclin-1, and p62 proteins, correlating with autophagic degradation [162].

### Fungicides

Thiram, a member of the dimethyldithiocarbamate (DDC) fungicide group, is used in rubber, plastic, and agricultural industries to protect crops and seeds from fungal infections [163]. Improper management or storage of thiram in chemical facilities and warehouses can lead to environmental contamination, given its widespread use in agriculture [163]. Prolonged exposure to thiram can cause sensitization and reproductive issues, as evidenced by altered gene expression in testicular cells, indicating potential autophagy induction via the mTOR/Atg5/p62 pathway, particularly at higher concentrations [164]. The recent study emphasizes that elevated thiram levels can adversely affect the testes by disrupting the BTB, leading to testicular tissue damage marked by decreased ZO-1 and Occludin mRNA expression, fibrosis promotion, and increased intercellular space [164]. Treatment with Thiram has also been demonstrated to elevate ROS production, reduce GSH levels, and induce oxidative stress [165].

## Intervention strategies for testicular injury mediated by environmental exposures

Currently, numerous antioxidants are utilized to ameliorate testicular injury by modulating oxidative stress or autophagy. In cases of testicular damage induced by environmental exposure through oxidative stress-mediated autophagy, therapeutic interventions typically employ various strategies to mitigate testicular harm: (1) through antioxidative mechanisms, involving the neutralization of free radicals, activation of antioxidant enzymes, and modulation of regulatory transcription factors, such as the Nrf2/HO-1 signaling pathway. (2) via anti-autophagic approaches, including the inhibition of autophagy-related proteins like Beclin-1 and LC3II, as well as modulation of signaling pathways influencing the autophagy process, such as the AMPK/mTOR signaling pathways.

### Antioxidants form a comprehensive defense mechanism against ROS

Antioxidant defense systems, incorporating enzymatic and non-enzymatic elements, serve to protect cellular and organ integrity from the deleterious impacts of free radicals. These antioxidants can either be endogenously synthesized or obtained from external sources like dietary intake or nutritional supplements [166]. Dietary and endogenous enzymatic and non-enzymatic antioxidants employ various strategies, such as electron donation, catalytic removal, or radical binding, as well as gene expression regulation, to counteract the deleterious impacts of free radicals [20].

1. **Free Radical Neutralization:** Antioxidants play a crucial role in neutralizing free radicals by donating electrons, thereby shielding against damage. Vitamin E, in its various forms, exhibits potent antioxidant properties by neutralizing lipid peroxyl radicals through hydrogen provision from the phenolic group on the chromanol ring [167]. Vitamin E can potentially mitigate the surge in free radical production induced by acrylamide in testicular tissues [168]. Additionally, Vitamin E treatment significantly reduces the impact of formaldehyde exposure on testicular structure, sperm quantity, and quality, attributed to its direct free radical scavenging abilities and interaction with membrane phospholipid bilayers to halt ROS-initiated chain reactions [90, 91]. Additionally, the hydroxyl group within the structure of carvacrol also contributes to mitigating spermatid differentiation disorders by reducing sodium arsenite-induced oxidative stress, inflammation, apoptosis, and autophagy [169].
2. **Activation of Antioxidant Enzymes:** Enzymes like SOD, CAT, glutathione peroxidase (GPx), among others, are integral components of the antioxidant defense system [170]. Quercetin, an antioxidant, effectively counteracts the decrease in activities of GSH, SOD, and GPx induced by cadmium exposure [171]. Furthermore, Açai berry demonstrates a significant elevation in Nrf2 and HO-1 levels, thereby enhancing the physiological antioxidant response by boosting CAT and GSH activity in cyclophosphamide-induced genitourinary damage [172].
3. **Regulatory Transcription Factor:** Nrf2 orchestrates antioxidant responses by activating defensive genes like heme oxygenase-1 (HO-1), glutathione-S-transferases, and NADPH quinone oxidoreductase 1, crucial for scavenging ROS and safeguarding cells from oxidative stress damage [173]. Studies indicate that azoramide treatment can inhibit the upregulation of Nrf2 induced by cadmium, hinting at its potential to suppress ROS production and mitigate cadmium induced mitochondrial injury [174]. Açai berry has also been shown to elevate Nrf2 and HO-1 levels in testes [172]. Eugenol exhibits the ability to reduce acrylamide induced ROS overproduction by facilitating testicular Nrf2 nuclear translocation and AKT phosphorylation, contrasting the effects observed in the acrylamide challenged group [133]. Additionally, the administration of Naringin holds promise in improving testicular damages by reversing the expression levels of p53, MAPK14, Caspase-3, and Bax proteins [132].

### Regulation of autophagy-related proteins and pathways

Proteins implicated in autophagy play distinct roles in governing this cellular process. Beclin-1 and LC3 act as pivotal markers of autophagic flux. Nano‑selenium has demonstrated efficacy in mitigating cadmium-induced disruption of autophagy by modulating signaling pathways associated with Beclin-1 and LC3 in Leydig cells [46]. Lactoferrin significantly alleviated spermatogenetic dysfunction by reducing the heightened ratios of BAX/BCL2 and LC3II/LC3I, and p62 protein expression [106]. In rats treated with carvacrol, the autophagy and inflammation triggered by sodium arsenite in testes were notably diminished, attributed to the downregulation of biomarkers such as LC3, MAPK-14, NF-κB, TNF-α, IL-1β, iNOS, and COX-2 [170]. Some pharmaceutical agents can alleviate testicular damage by boosting autophagy, as indicated by the decrease in p62 level and LC3II/LC3I ratio in the cadmium+quercetin group, implying quercetin’s ameliorative effect on cadmium-induced autophagy [171]. Resveratrol treatment enhanced cell viability, SOD activity, and anti-apoptotic effects in nicotine-exposed Leydig cells, potentially offering cytoprotective benefits against oxidative damage through autophagy activation via the AMPK/mTOR pathway [87]. Meanwhile, eugenol treatment significantly enhanced sperm quality parameters through the improvement of ROS-mediated autophagy, apoptosis, and BTB remodeling [133]. Furthermore, sitagliptin, acting as a selective DPP4 inhibitor, can attenuate the activity of the ERK and AKT pathways while suppressing the AMPK signaling cascade, all of which are involved in autophagy regulation [175].

## Conclusion

In summary, this review consolidates existing literature on how environmental exposures contribute to testicular damage by disrupting cellular autophagy and oxidative stress balance, showcasing recent significant progress. Despite varying study designs and methodologies, a consensus is emerging on how environmental factors trigger oxidative stress by elevating ROS levels, impacting autophagy via pathways like ROS-mediated mTOR pathway, ultimately leading to testicular damage. Moreover, the interplay between autophagy and oxidative stress, particularly the relationship between p62 and Nrf2, underscores their crucial roles. Furthermore, certain drugs demonstrate the potential to mitigate testicular damage by modulating oxidative stress and autophagy. These discoveries emphasize the necessity of elucidating the mechanisms of autophagy influenced by environmental exposures in disrupting oxidative stress equilibrium, pinpointing drug targets, and laying a foundation for optimizing future treatments and clinical management of testicular injuries.
